# Effectiveness of preoperative pelvic floor muscle training for urinary incontinence after radical prostatectomy: a meta-analysis

**DOI:** 10.1186/1471-2490-14-99

**Published:** 2014-12-16

**Authors:** Wei Wang, Qing Mei Huang, Feng Ping Liu, Qi Qi Mao

**Affiliations:** Nursing Education Center, The First Affiliated Hospital, School of Medicine, Zhejiang University, 79 Qingchun Road, Hangzhou, Zhejiang 310003 People’s Republic of China; Department of Urology, The First Affiliated Hospital, School of Medicine, Zhejiang University, 79 Qingchun Road, Hangzhou, Zhejiang 310003 People’s Republic of China

**Keywords:** Pelvic floor muscle training, Urinary incontinence, Prostate cancer, Meta-analysis

## Abstract

**Background:**

Radical prostatectomy (RP) is the most common treatment for patients with localized prostate cancer. Urinary incontinence (UI) is a significant bothersome sequela after radical prostatectomy that may dramatically worsen a patient’s quality of life. Pelvic floor muscle training (PFMT) is the main conservation treatment for men experiencing urinary incontinence; however, whether additional preoperative PFMT can hasten the reestablishment of continence is still unclear. The objective of this meta-analysis is to determine whether the effectiveness of preoperative plus postoperative PFMT is better than postoperative PFMT only for the re-establishment of continence after RP.

**Methods:**

A meta-analysis was performed after a comprehensive search of available randomized controlled trials (RCTs). Quality of the included studies was assessed by the Cochrane Risk of Bias tool. Efficacy data were pooled and analyzed using Review Manager (RevMan) Version 5.0. Pooled analyses of continence rates 1, 3, 6, and 12 months postoperatively, using relative risk (RR) and 95% confidence intervals (CIs), were conducted. For data deemed not appropriate for synthesis, a narrative overview was conducted.

**Results:**

Five eligible studies were ultimately included in this analysis. No significant differences in continence rates were detected at the early (1- and 3-month) time points: RR = 1.21, 95% CI = 0.71–2.08, *P* = 0.48; RR = 1.1, 95% CI = 0.09–1.34, *P* = 0.34, respectively), interim (6-month time point: RR = 0.98, 95% CI = 0.93–1.04, *P* = 0.59), or late recovery stage (RR = 0.93, 95% CI = 0.67–1.29, *P* = 0.66). Outcomes reported were time to continence in two trials and quality of life in three, but results were inconclusive because of insufficient data.

**Conclusion:**

According to this meta-analysis, additional preoperative PFMT did not improve the resolution of UI after RP at early (≤3-month), interim (6-month), or late (1-year) recovery stages. However, the results of time to continence and quality of life were inconclusive because of insufficient data. More high-quality RCTs are needed for better evaluation of the effectiveness of preoperative PFMT on post-prostatectomy UI.

## Background

Radical prostatectomy (RP) is the most common treatment for patients with localized prostate cancer [1]. In addition to traditional open RP, currently more advanced surgical methods, such as robot-assisted laparoscopic RP and laparoscopic RP, are widely used. However, despite improvements in surgical technique, urinary incontinence (UI) remains a significant and bothersome sequela after RP that may dramatically worsen a patient’s quality of life [2–4]. UI after RP has been attributed to urethral sphincter deficiency or injury, and to bladder dysfunction such as detrusor overactivity, impaired bladder-filling sensation, and low bladder compliance [5]. Therapeutic strategies for UI include conservative treatment, pharmacotherapy, penile clamping, and artificial urinary sphincter [6]. A conservative treatment approach to UI has the advantage of noninvasiveness. The theoretical basis of pelvic floor muscle training (PFMT) is that repeated volitional contractions of selected pelvic floor muscles may improve their strength and efficiency during periods of increased intra-abdominal pressure, and PFMT has been shown to be effective in relieving stress UI in women [7]. However, despite the popularity of PFMT as conservation treatment for UI, a Cochrane review concluded that there was insufficient evidence to show whether it was effective or not [8]. The reason may be the duration of PFMT was not long enough**.** Actually, many studies reported that their study patients started PFMT after catheter removal [9–12]. However, others investigated the effects of preoperative PFMT on the duration and severity of UI for patients with RP and had positive results [13–16]. But a recent randomized controlled trial (RCT) in which patients started PFMT 3 weeks before surgery [17] had results that were inconsistent with other studies, and another RCT, in which patients began PFMT with biofeedback 4 weeks before surgery even appeared to show greater benefit for the control group [18]. These contradictory results may be attributable to sample size and/or RCT quality. It is therefore essential to determine whether patients with additional preoperative PFMT regain urinary continence earlier than patients with only postoperative PFMT after RP. As such, we intended to provide the best available evidence through a strict meta-analysis. We hypothesized that additional preoperative PFMT would have a positive effect on urinary incontinence after RP, at least at the early recovery stage.

## Methods

### Literature search

**Figure 1:**
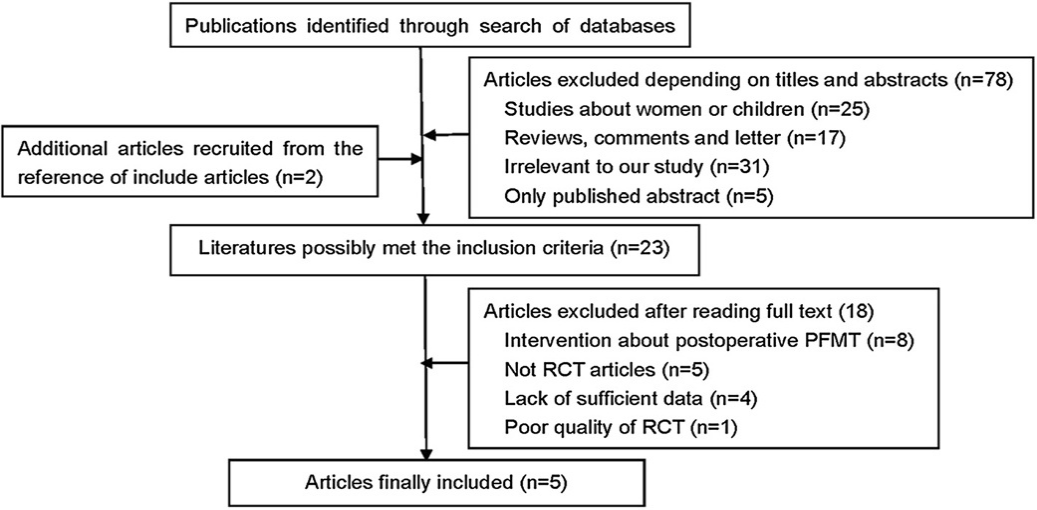
**Flow diagram of article selection.**

A comprehensive and systematic search was conducted to obtain a full view of the influence of preoperative and postoperative PFMT compared with only postoperative PFMT on UI after RP. Search terms *preoperative*, *pelvic floor muscle training* or *pelvic floor muscle exercise,* and *radical prostatectomy* were used to search publications dated through July 2014. We first searched the Cochrane Library for reference lists of registered RCTs and reviews, and then searched the PubMed database and Web of Science to identify available publications. Additional publications were also searched from references cited in retrieved articles. Publication language was restricted to English. Eligible articles were included in the meta-analysis if the following criteria were met: 1) Studies were RCTs or quasi-RCTs evaluating the effectiveness of preoperative and postoperative PFMT compared with postoperative PFMT on UI after RP; 2) surgical method was open radical prostatectomy, robot-assisted laparoscopic radical prostatectomy, or laparoscopic radical prostatectomy; 3) intervention was PFMT with or without biofeedback, physiotherapist guidance, or electrical stimulation; and 4) outcomes were number or percentage of patients recovering continence, time to continence, and quality of life. Studies with only abstracts or with insufficient data were excluded. Figure 1 is a flow chart of the search and identification of articles.

### Quality evaluation and data extraction

The quality of the included RCT was assessed by using the Cochrane Risk of Bias tool [19]. The following six methodological parameters were evaluated, including 1) Identification of study as RCT or quasi-RCT (method of random sequence generation); 2) comparison of baseline data between experiment and control groups; 3) concealment of treatment allocation for randomization; 4) whether blinding, mainly of the outcome assessor was implemented; 5) whether dropouts were reported; and 6) intention-to-treat analysis. Quality of the included studies was ranked as A (a study with low risk of bias), B (medium risk of bias), or C (high risk of bias). Studies ranked C were excluded because of a high risk of bias. The quality of included studies is shown in Table 1.

**Table 1:** **Quality of eligible studies**

Authors, year	Randomization	Baseline	Allocation concealment	Blinding	Loss to follow-up	Intention-to-treat analysis	Quality rank ^a^
Bales et al., 2000 [20]	Unclear	Comparable	Unclear	Yes	Reported	Unclear	B
Centemero et al., 2010 [16]	Computer	Comparable	Yes	Yes	Reported	Yes	A
Dijkstra-Eshuis et al., 2013 [18]	Computer	Comparable	Yes	Yes	Reported	Yes	A
Geraerts et al., 2013 [17]	Computer	Comparable	Unclear	Yes	Reported	Yes	B
Patel et al., 2013 [21]	Date of surgery	Comparable	Unclear	Unclear	Reported	Yes	B

^a^A, low risk of bias; B, medium risk of bias; C, high risk of bias.

Data extraction from qualified articles was conducted by two authors independently using a standard form developed for this purpose. Information extracted included first author’s name, publication year, study design, sample size, intervention method, duration of preoperative PFMT, definition of continence, and outcomes. To ensure completeness and accuracy of the extracted data, the two authors compared and cross-checked their tasks, and disagreement was resolved by discussion. Table 2 presents the characteristics of eligible studies.

**Table 2:** **Characteristics of eligible studies**

Authors, year	Design	Sample size (E/C)	Preoperative PFMT method	Time started PFMT before surgery	Definition of continence	Outcomes	Data (E vs. C)			
							1 month	3 months	6 months	12 months
Bales et al., 2000 [20]	RCT	100 (47/50)	PFMT with biofeedback four times per day	2–4 weeks	Defined as the use of one or more pads per day	Urinary continence (assessed by a single nurse)	9 vs.12	27 vs. 31	44 vs. 48	(6-month F/U)
Centemero et al., 2010 [16]	RCT	118 (59/59)	PG-PFMT by a single physiotherapist	30 days	Defined as the sum of no urinary leakage	Self-reported continence	26 vs. 12	35 vs. 22	(3-month F/U)	
						QoL score (ICS male SF)	Difference is significant at both 1- and 3-month time points, suggesting preoperative PFMT may improve QoL			
Dijkstra-Eshuis et al., 2013 [18]	RCT	121 (65/56)	PFMT with biofeedback once a week for 30 min	4 weeks	Defined as no leakage at all	Continence (24-h pad test)	Only 12-month- time-point data were available for extraction			38/58 vs. 36/45
						QoL (measured by KHQ and IPSS)	No significant difference at each time point			
Geraerts et al., 2013 [17]	RCT	180 (91/89)	PG-PFMT 30 min per week	3 weeks	Defined as 3 consecutive days of 0 g of urine loss on a 24-h pad test	Incidence of continence	44/86 vs. 44/87	67/86 vs.71/87	80/86 vs. 80/85	83/85 vs. 81/85
						Time to continence (24-h pad test)	Median times to continence were 30 and 31 days for the C and E groups, respectively (*P* = 0.878)			
						QoL (measured by KHQ)	No difference at any time point, except in one aspect of the KHQ at 3 and 6 months (*P* = 0.008 and *P* = 0.024, respectively)			
Patel et al., 2013 [21]	Quasi-RCT	284 (152/132)	PG-PFMT	4 weeks	Use of zero to one pad	Continence percent (24-h pad weight)	6 weeks 25% (38) vs. 17% (23)	73% (112) vs. 62% (82)	(3-month F/U)	
						Time to achieve continence	Preoperative PG-PFMT is effective in reducing time to continence			

C = control group, E = experimental group, F/U = follow-up, ICS male SF = International Continence Society male short form, IPSS = International Prostate Symptom Score, KHQ = King’s Health Questionnaire, PG-PFMT = physiotherapist-guided pelvic floor muscle training, QoL = quality of life, RCT = randomized controlled trial.

### Statistical analysis

Efficacy data were pooled and analyzed using Review Manager (RevMan) Version 5.0 The primary outcome was number or percentage of patients who achieved urinary continence. For dichotomous outcomes, risk ratio (RR) and 95% confidence interval (CI) were applied to evaluate the effectiveness of additional preoperative PFMT for UI after RP at each follow-up time point. Heterogeneity between eligible studies was assessed using chi^2^ and the *I*^*2*^ test. Heterogeneity was considered significant for *P* < 0.1 or *I*^*2*^ > 50% [22–24]. *I*^*2*^ was the percentage of variation attributed to the heterogeneity, which was considered low if *I*^*2*^ value was less than 50%. Data were pooled and analyzed using either the fixed-effects or random-effects model depending on the results of the calculation of heterogeneity. If there was no or low heterogeneity (*I*^*2*^ *<* 50%), the fixed-effects model was selected. Because there were only five qualified studies, publication bias was not assessed. For the subordinate outcomes in our study, including mean time to continence and quality-of-life scores, data for which were deemed inappropriate for synthesis because of a great deal of heterogeneity between-studies, a narrative overview was implemented. Sensitivity analysis was conducted by sequential removal of each study and then recalculation of the pooled estimates for the remaining studies, with the aim of verifying the reliability of the result.

### Ethical approval and consent

Ethical approval was obtained from the ethics committee of The First Affiliated Hospital, College of Medicine, Zhejiang University. The requirement for informed consent was waived.

## Results

### Eligible studies

Only five published studies [16–18, 20, 21] comparing the effectiveness of preoperative and postoperative PFMT, with that of only postoperative PFMT on UI met the inclusion criteria. Four of them were RCTs [16–18, 20] and one was a quasi-RCT [21]. Baseline data for all included studies were comparable between the intervention and control groups, and each study reported dropouts. Analysis by intention-to-treat principle was clearly declared, except in one trial [20]. After risk of bias of the five studies was assessed using the Cochrane Risk of Bias tool [19], two trials [16, 18] were ranked A and the other three [17, 20, 21] were ranked B (Table 1). Of the five, two trials [18, 20] implemented PFMT with biofeedback as preoperative intervention, whereas three trials [16, 17, 21] used physiotherapist-guided PFMT. PFMT was started 2–4 weeks before surgery. Study follow-up duration was between 1 and 12 months. Data were extracted and pooled at each time point (1, 3, 6, and 12 months) and were categorized by the reviewers as early (1- and 3-month), interim (6-month), and late (12-month) stages of resolution of UI after RP.

### Statistical analysis

#### Effectiveness of preoperative PFMT for urinary continence at different time points

**Figure 2:**
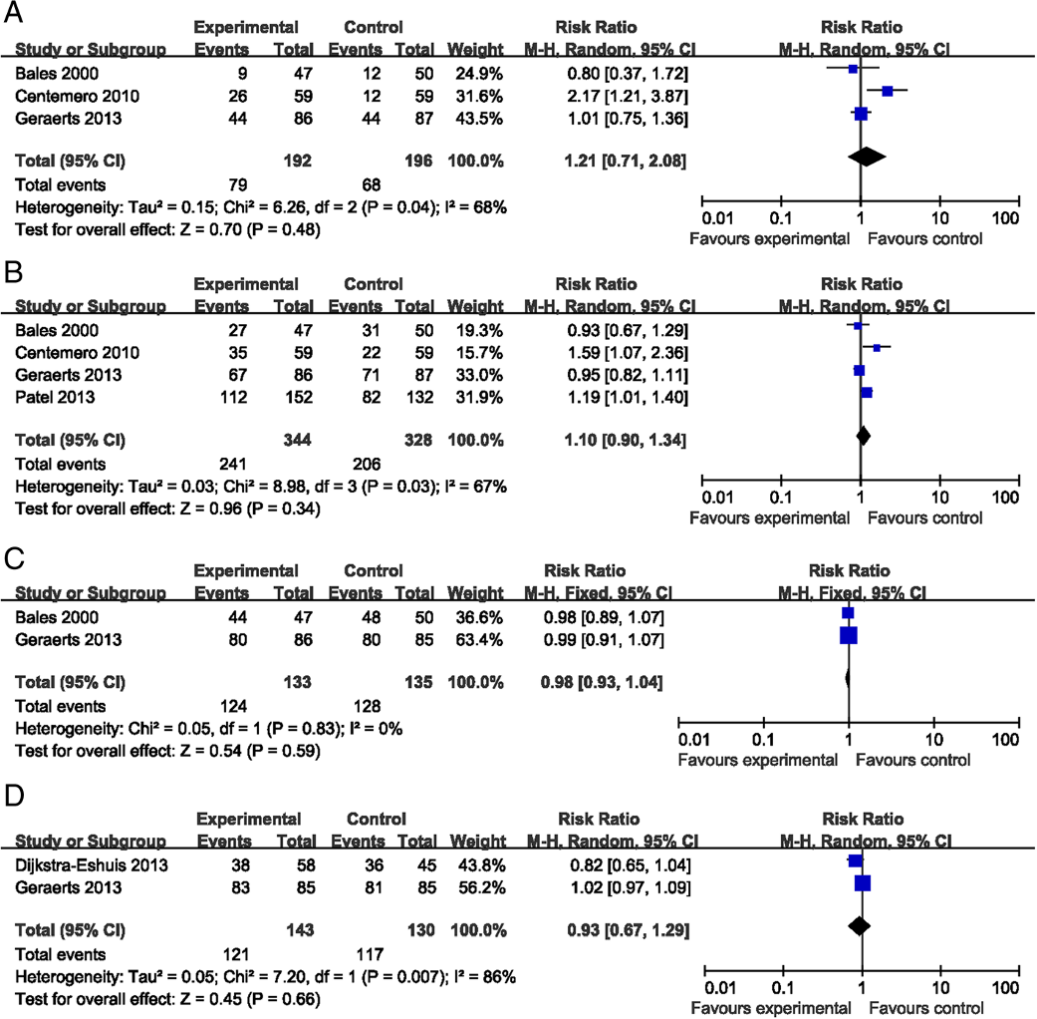
**Forest plots depicting the effectiveness of additional preoperative PFMT for post-prostatectomy urinary incontinence at different time points. (A)** Pooled analysis of three eligible studies at the 1-month time point using a random-effects model. **(B)** Pooled analysis of four eligible studies at the 3-month time point using a random-effects model. **(C)** Pooled analysis of two eligible studies at the 6-month time point using a fixed-effects model. **(D)** Pooled analysis of two eligible studies at the 12-month time point using a random-effects model.

Three trials [16, 17, 20] enrolling 388 patients (intervention group = 192, control group = 196) reported the continence rate at 1 month after RP. There was high heterogeneity between studies (*I*^*2*^ = 68%, *P* = 0.04 < 0.1); therefore, the random-effects model was selected. One trial reported that the percentage of patients who were continent at 1 month was significantly higher in the intervention group (44.1% vs. 20.3%, *P* = 0.018). However, the pooled-effect size showed no significant difference between the intervention and control groups (RR = 1.21, 95% CI = 0.71–2.08, *P* = 0.48). Four studies [16, 17, 20, 21] involving 672 patients (intervention group 344, control group 328) provided data at the 3-month time point; therefore, a random-effects model was used based on the heterogeneity test (*I*^*2*^ = 67%, *P* = 0.03 < 0.1); pooled analysis did not show a relative benefit for the additional preoperative PFMT group (RR = 1.1, 95% CI = 0.09–1.34, *P* = 0.34). In addition, pooled analysis also showed a non-significant difference at the 6-month [17, 20] and 12-month [20, 21] time points (RR = 0.98, 95% CI = 0.93–1.04, *P* = 0.59; RR = 0.93, 95% CI = 0.67–1.29, *P* = 0.66, respectively). These results did not support our hypothesis. This meta-analysis of five qualified RCTs indicated that additional preoperative PFMT did not improve the reestablishment of urinary continence after radical prostatectomy (Figure 2). Sensitivity analysis did not greatly alter the results at each time point.

#### Effectiveness of preoperative PFMT in reducing time to continence and improving quality of life

Two trials [17, 21] reported time to continence as an outcome. However, because of the variability in methods of measuring outcome and in definition of continence, the data for which were deemed not inappropriate for synthesis, so a narrative overview is presented. Geraerts et al. [17] used the 24-h pad test to measure time to continence, which was strictly defined as 3 consecutive days of 0 g of urine loss. He concluded that the median times to urinary continence, 30 days for the control group and 31 days for the experimental group, were approximately the same (*P* = 0.878). Patel et al. [21] defined time to achieve continence as zero pads used per day, by patient report, and demonstrated a median of 8 weeks and 7 weeks to continence for the control and intervention groups, respectively. This was a low significant difference (*P* = 0.047), but the authors also indicated that multivariable Cox regression analysis of preoperative PFMT, when acting as the independent factor affecting time to achieve continence, revealed no significant difference between groups (*P* = 0.084). Similarly, the outcome of quality of life was assessed by three trials [16–18]. One trial [16–18] used the International Continence Society male short form to assess participants’ quality of life and drew a cautious conclusion that preoperative PFMT may improve quality of life at 1- and 3-month time points. However, the other two trials [17, 18] used the King’s Health Questionnaire to evaluate the impact of UI on quality of life. One trial [18] revealed there were no significant differences in quality of life between the intervention group and the control group at each postoperative time point (6 weeks, 3 months, 6 months, 9 months, and 1 year), while the other one [17] reported that the intervention group had better King’s Health Questionnaire scores 3 and 6 months after surgery. Our meta-analysis was therefore unable to provide a consistent conclusion regarding the outcome of time to continence and quality of life because of a lack of appropriate data.

## Discussion

Our meta-analysis attempted to clarify the controversial issue of whether additional preoperative PFMT would hasten the resolution of urinary incontinence after RP. After a comprehensive search of electronic databases and strict assessment of quality, five RCTs were deemed qualified for inclusion. However, the pooled analysis suggested no benefit from additional preoperative PFMT for UI after RP at 1- and 3-, 6-, and 12-month time points, which represented early, interim, and late recovery phases in the present analysis.

Urinary incontinence is most severe in the early postoperative phase; therefore, two of the five included studies [16, 21], which were conducted to determine the effectiveness of preoperative PFMT in the early recovery stage (within 3 months) indicated significantly decreased duration and severity of early urinary incontinence after RP. These positive results formed the foundation of our hypothesis. However, results of the pooled analysis were inconsistent with our hypothesis and indicated there was no benefit from additional preoperative PFMT, even at the very early stage of recovery (within 1 month). It must be pointed out, however, that the findings should be interpreted with caution because of the unavailability of evidence regarding additional preoperative PFMT and considerable between-study clinical heterogeneity, one aspect of which is the different durations of preoperative exercise. Two trials [16, 21], which started PFMT 4 weeks before surgery, had findings in favor of the experimental group, whereas two other trials commenced exercise 3 weeks [17, 20] and 2–4 weeks [17, 20] before surgery but did not find a significant benefit from preoperative PFMT. The inconsistent results may be because of the different durations of preoperative exercise; it can be assumed that while a short duration of exercise provides time for awareness of the pelvic floor muscles, a longer preoperative exercise period to enhance strength and endurance would provide greater benefit [17]. Another critical issue is the frequency of PFMT. Novara [25] points out that the greater the frequency of PFMT, the better its efficacy, regardless of the method chosen.

Considering the variation among studies and that the data for time to continence and quality of life were insufficient for a pooled analysis, we provided only a narrative description of the secondary outcomes. Outcomes between different trials were conflicting, and this meta-analysis was unable to provide a consistent conclusion. The two trials [17, 21] reporting the outcome of time to continence used different measurements and definitions of continence, and produced entirely different results. Geraerts et al. [17] indicated that the median time to continence was almost the same for both groups, while Patel et al. [21] found that a PFMT program begun preoperatively could significantly reduce the duration of early incontinence. More evidence is needed to corroborate these results. Similarly, the outcome of quality of life reported by three trials [16–18] will also require further investigation to resolve these inconsistent results. Geraerts et al. [17] found the impact of incontinence on quality of life to be smaller in the preoperative-exercise group but no explanation was given for the better quality-of-life scores obtained by the experimental group. The authors also evaluated satisfaction with preoperative PFMT, and all patients in the experimental group expressed satisfaction at receiving PFMT before surgery despite the lack of reduction in their postoperative duration of incontinence. The study by Centemero et al. [16] showed that 75% of patients in the intervention group reported a high degree of satisfaction with starting PFMT before surgery. Considering that UI is a complication that causes patients a great deal of stress and has a particularly negative impact on quality of life, any intervention that can shorten its duration is worth trying. More importantly, men receiving additional PFMT before surgery showed a high degree of satisfaction, so we still recommend PFMT, with its advantage of noninvasiveness, as the main strategy for prevention and treatment of UI after RP even though our meta-analysis did not clearly demonstrate a benefit.

As is often the case with a meta-analysis, our study has several limitations that could bias the final results. First, we included only five studies, based on our strict inclusion criteria, which was not enough to draw a strong conclusion. Second, funnel plots were not created to assess publication bias because of the limited available evidence; we merely included the studies published in English, which may widen the publication bias and affect the analysis. The third major limitation was the clinical heterogeneity caused by non-standardized treatment regimens and outcome measures. Treatment regimens varied between studies in surgical technique, frequency of PFMT (four times or 30 min daily), definitions of continence and incontinence, durations of follow-up (3, 6, or 12 months), timing of initiation of preoperative PFMT (2–4 weeks before surgery), and outcome measurements (24-h pad test, 1-h pad test, different questionnaires). For example, in our meta-analysis, the surgical techniques included open prostatectomy, and laparoscopic and robit-assisted laparoscopic radical prostatectomy, however, because of an insufficient number of qualified RCTs, we could not perform a subgroup analysis by category of surgical technique, which was a source of bias affecting the results. It is noteworthy that factors associated with resolution of UI are complicated and include a patient’s age, tumor stage, and history of previous lower urinary tract dysfunction [26]. These probably also decrease the credibility and validity of the results of this meta-analysis.

## Conclusion

Our meta-analysis suggests that additional preoperative PFMT did not improve the rate of reestablishment of continence after RP at the early (within 3 months), interim (6-month), or late (1-year) stage. However, we were unable to draw a definitive conclusion regarding the outcomes of time to continence and quality of life. Further high-quality RCTs with larger samples, standardized treatment regimens, and credible outcome measurements are warranted to confirm or refute our results.

## Authors’ information

Wei Wang, PhD, Director of Nursing Education Center, Head Nurse of Department of Urology, The First Affiliated Hospital, School of Medicine, Zhejiang University.

Qing Mei Huang, BSN, Nursing graduate student of Nursing Education Center, The First Affiliated Hospital, School of Medicine, Zhejiang University.

Feng Ping Liu, MSN, doctoral student of nursing, Nursing Education Center, The First Affiliated Hospital, School of Medicine, Zhejiang University.

Qi Qi Mao, MD, PhD; Attending Physician, Department of Urology, The First Affiliated Hospital, School of Medicine, Zhejiang University.

Wei Wang and Qing Mei Huang are co-first authors.

## Abbreviations

CI: Confidence interval
PFMT: Pelvic floor muscle training
RCT: Randomized controlled trial
RP: Radical prostatectomy
RR: Relative risk
UI: Urinary incontinence.
